# Assessment of Airway Changes During Pregnancy With Ultrasonography and Clinical Airway Screening Tests: A Prospective Observational Study

**DOI:** 10.7759/cureus.83938

**Published:** 2025-05-12

**Authors:** Madhuri Kurdi, Bhuvanvijay D, Kaushic A Theerth, Jagadish G Sutagatti, Kavya Ananthathirtha

**Affiliations:** 1 Anaesthesiology and Critical Care Medicine, Karnataka Medical College and Research Institute, Hubballi, IND; 2 Anaesthesiology and Critical Care, Medical Trust Hospital, Ernakulam, IND; 3 Radiodiagnosis, Karnataka Medical College and Research Institute, Hubballi, IND; 4 Obstetrics and Gynaecology, Neighborhood Hospital, Bangalore, IND

**Keywords:** airway ultrasound, modified mallampati class, neck circumference, pathophysiological changes in pregnancy, three trimesters of pregnancy

## Abstract

Introduction: Ultrasonographic airway assessment is a promising adjunct to predict difficult intubation; however, it has been relatively less studied in obstetric cases. This study aimed to assess the progress of anatomical changes in the obstetric airway, through all the three trimesters, using ultrasound measurements and clinical screening methods.

Materials and methods: This prospective observational study included 205 women with singleton pregnancies. In clinical assessment, modified Mallampati class and neck circumference were noted. In ultrasonographic assessment, the skin-to-epiglottis distance and tongue volume were measured. In every study participant, the measurements were recorded three times, once in each of the three trimesters. The primary objective was to assess and compare the clinical and ultrasonographic airway changes during the three trimesters of pregnancy. Secondary objectives were to assess airway changes among those with pre-eclampsia and to assess clinical parameters, such as history of snoring, change of voice, and presence of facial oedema throughout the pregnancy.

Results: There was an increase in the modified Mallampati grade (p = 0.0001), neck circumference (mean difference = -0.40, p = 0.0001), skin to epiglottis distance (mean difference = -0.82, p = 0.0001), and tongue volume (mean difference = -0.84, p = 0.0001), especially during the third trimester.

Conclusion: During the course of normal pregnancy, the skin-to-epiglottis distance and tongue volume increased progressively through the trimesters, reflecting the changes in the Mallampati scores and neck circumference. These findings could help in the prediction and management of the difficult airway during various stages of pregnancy.

## Introduction

Preoperative assessment of the airway is particularly important in obstetric anaesthesia because failed airway management is one of the leading causes of maternal morbidity and mortality [1]. The physiological changes in the airway that occur in various trimesters are the main contributors to the failed or difficult airway. The mucosal changes in the upper airway include hyperaemia, oedema due to leakage of plasma into the stroma, glandular hypersecretion, increased phagocytic activity, and increased mucopolysaccharide content [2]. The common clinical methods used to assess airway changes include Mallampati classification, hyomental and thyromental distance, neck movements, inter-incisor distance, and neck circumference [3]. However, their sensitivity and specificity for the accurate prediction of a difficult airway are not very robust in obstetric cases [4,5]. Point-of-care ultrasound (POCUS) of the airway is capable of providing detailed anatomical information and is used to identify airway pathology [6]. Nowadays, POCUS of the airway has been recommended (though a weak level of recommendation) as a routine preoperative investigation [7].

The obstetric airway is an entity that needs further research. Not many researchers have endeavoured to conduct research on the obstetric airway mainly due to ethical issues. Nevertheless, an extensive literature search revealed a scarce number of studies on this subject. Ultrasound of the obstetric airway is an even less touched-upon topic. The ultrasonographic parameters have been assessed by a few researchers in the third trimester of pregnancy, during labour, and in the immediate postpartum period [8]. We assessed the airway changes that occur in normal pregnancy over the three trimesters using ultrasonographic and clinical parameters. No other researchers, as per our knowledge, have assessed these changes over the three trimesters by ultrasonography.

This study aimed to assess the progress of airway changes throughout the various trimesters using clinical and ultrasonographic parameters. The primary objective of the study was to assess and compare the airway changes during the three trimesters of pregnancy using ultrasonographic measurement of anterior neck soft tissue thickness (using skin to epiglottis distance) and tongue volume and clinical screening methods such as Mallampati scoring and neck circumference measurement. Secondary objectives were to assess airway changes among special patient groups, such as those with pre-eclampsia, and assessment of clinical parameters, such as history of snoring during sleep, change of voice, and presence of facial oedema throughout the pregnancy.

## Materials and methods

This prospective observational study was conducted on pregnant patients aged between 18 and 45 years at a tertiary healthcare institute from May 2023 to May 2024. After obtaining Institutional Ethics Committee approval (318:2022-23) and registration with the Clinical Trials Registry of India (CTRI/2023/05/053171), the study was carried out in accordance with the principles of the Declaration of Helsinki, 2013, and good clinical practice. Pregnant women visiting the antenatal clinic (ANC) for regular checks were included, and their age, weight, height, gestational age, blood pressure, and body mass index (BMI) were recorded. Patients having a BMI >35 kg/m^2^, anatomical abnormalities of the face and neck, and any thyroid swelling/thick scars over the anterior neck were excluded from the study. The patients were informed about the procedure in detail, and consent was obtained. We noted and recorded the presence of pregnancy-induced hypertension (PIH) in the study cases. PIH was defined as pregnancy with high blood pressure (≥140/90 mmHg) after 20 weeks of gestation, measured two times, six hours apart, with or without proteinuria [9]. The diagnosis of PIH was confirmed by the obstetrician working in the ANC. The patients were then referred to the pre-anaesthesia examination clinic by the obstetrician.

Clinical assessment included modified Mallampati (MMP) score and neck circumference measurements. For determining the MMP score, the patient was assessed in the sitting posture with the head in a neutral position. They were asked to fully open the mouth and protrude the tongue maximally without phonating and the grading was done (Grade: Class 1: soft palate, fauces, uvula, and pillars visible; Class 2: soft palate, fauces, and uvula visible; Class 3; soft palate and base of uvula visible; Class 4: none of the soft palate visible). The neck circumference was measured with the patient in the upright and seated position at the level of the thyroid cartilage using a standard tape and recorded in centimetres.

**Figure 1:**
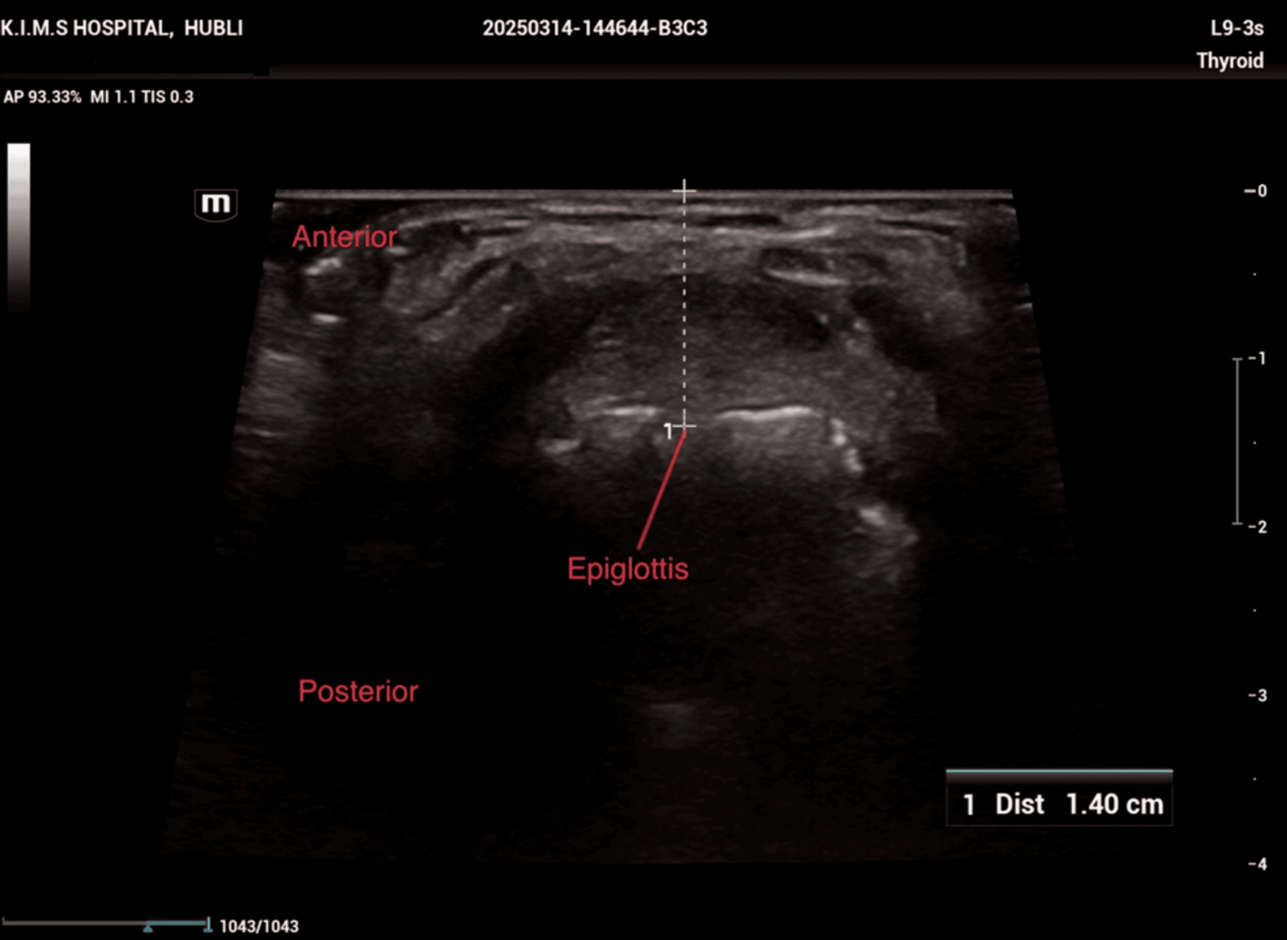
Ultrasonography measuring the skin-to-epiglottis distance

Ultrasonographic airway parameters were measured by an anaesthesiologist experienced in ultrasonography of the upper airway. The patients were made to lie in the supine position with a wedge under the right buttock and head in the neutral position without a pillow, looking straight ahead with the mouth closed and the tongue lying still on the floor of the mouth. An ultrasound machine (Philips, Bothell, WA) with linear (4-12 MHz) and curvilinear (2-7 MHz) transducers was used to measure the different ultrasonographic parameters. The probes were placed on the skin under the patient’s chin, at different levels, to get a transverse view of the submandibular area and the upper part of the neck. The transverse view was obtained using a linear transducer for measuring the skin to epiglottis distance in centimetres at the level of the thyrohyoid membrane (Figure 1).

**Figure 2:**
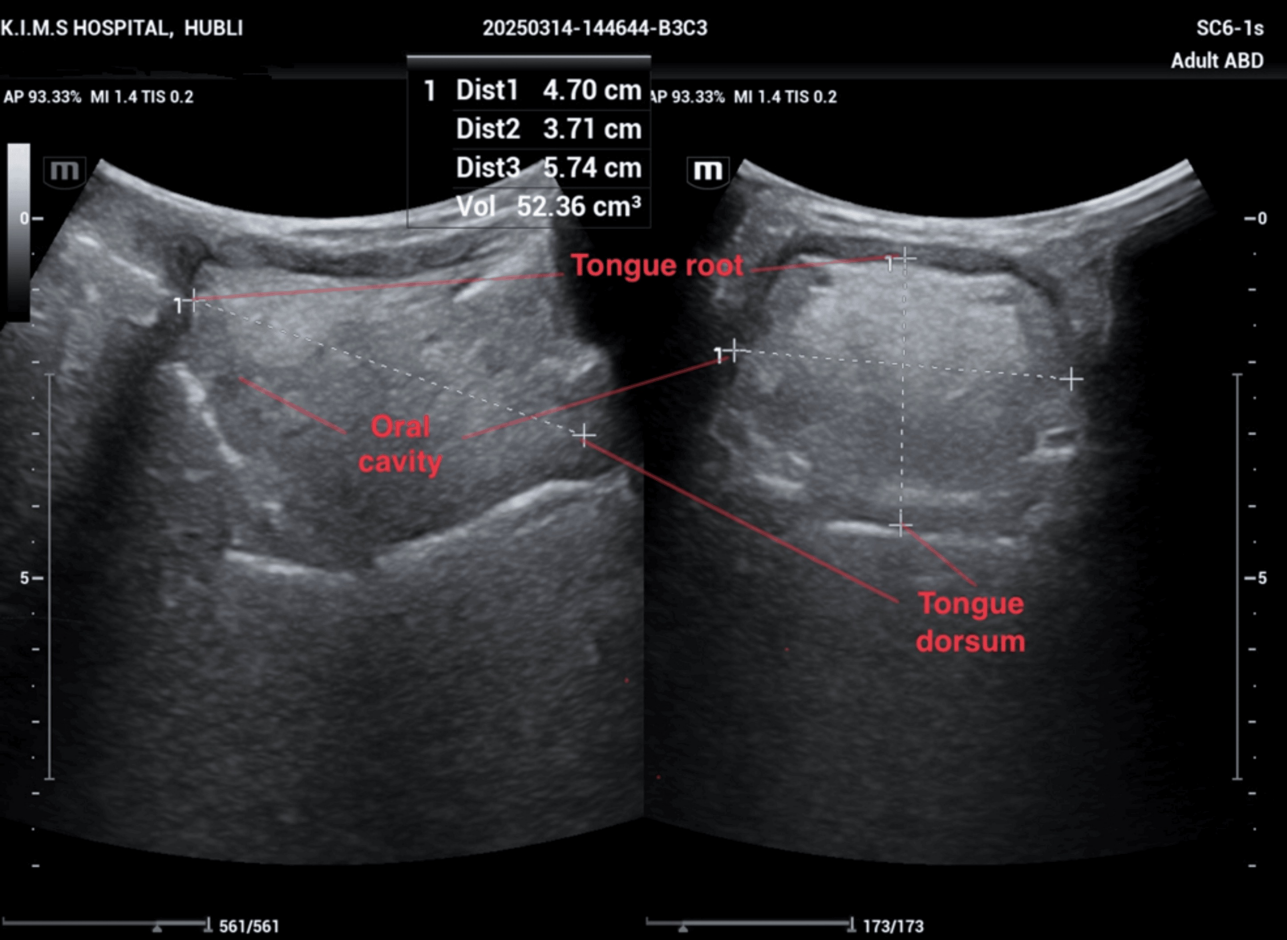
Ultrasonographic measurement of the tongue volume

The tongue volume (Figure 2) was obtained by multiplication of the midsagittal cross-sectional area and width in the transverse plane with the help of a curvilinear and linear transducer, respectively [3,10].

The measurements were taken three times in a patient, at specific time points in each of the three different trimesters during the course of pregnancy. At any given time, two ultrasonographic measurements were taken, and the average value was entered. Assessment was done at the time of regular ANC visit to the obstetric outpatient department, which included first trimester up to 12 weeks of gestational age, second trimester up to 26 weeks, and third trimester up to 40 weeks of gestational age. The airway scans were made to coincide with the obstetric scans and performed simultaneously for logistical convenience. The incidence of clinical parameters such as history of snoring, change of voice, and presence of facial oedema throughout the pregnancy was recorded.

 In a previous study conducted by Kodali et al. [8], the MMP airway class increased by either one grade or two grades through the pregnancy in 38% of women. Considering the proportion of pregnant women who would experience a change in airway class during the three trimesters of pregnancy to be around 38%, the sample size was calculated as 185 by using an α error of 0.05(5%) and a d (absolute error) of 7%. Considering a 10% loss of follow-up, a sample size of 185 + 18.5 ≈ 205 pregnant women was finally decided for the study.

The data collected were entered in a Microsoft Excel sheet (Microsoft® Corp., Redmond, WA) and analyzed using Statistical Package for Social Sciences softwareStatistical Product and Service Solutions (SPSS, version 25.0; IBM SPSS Statistics for Windows, Armonk, NY). Continuous data were represented as mean and standard deviation (SD). Dependent t-test, McNemar test, Cochran Q test, Friedman test, and Wilcoxon matched pairs test were used for data analysis. A p-value <0.05 was considered significant.

## Results

A total of 205 pregnant women were included in the study, with a mean (SD) age of 24 (2.76) years and a height of 155 (3.69) cm (Table 1). 

**Table 1:** Demographic variables n - number

Variables (n=205)	Minimum	Maximum	Mean	Standard deviation
Age in years	20.00	32.00	24.00	2.76
Height in cm	148.00	165.00	155.90	3.69

**Figure 3:**
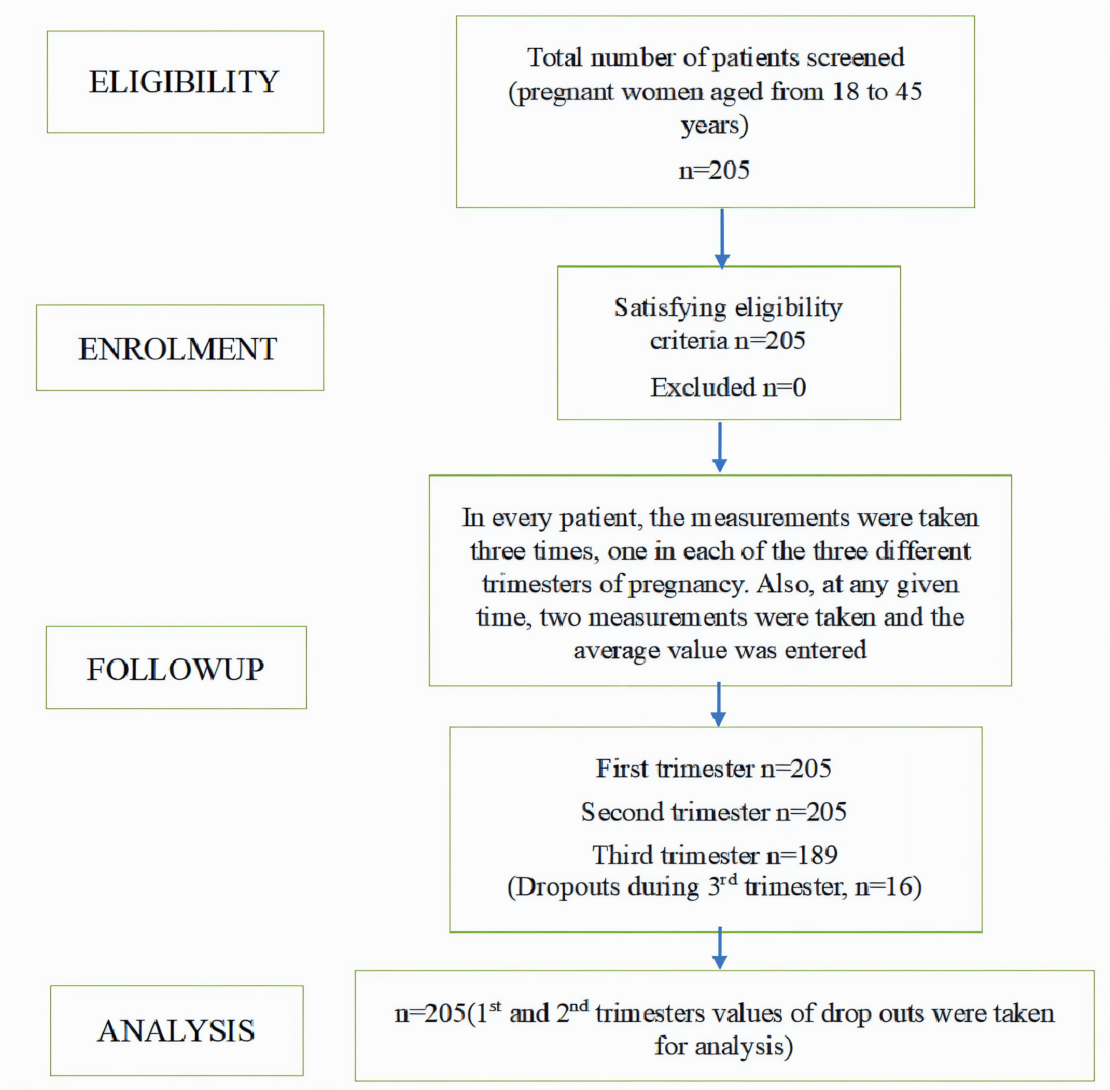
STrengthening the Reporting of OBservational studies in Epidemiology (STROBE) flow diagram n - number

All the included patients were singleton pregnancies. The same number of patients were examined in the first and second trimesters, while the third trimester had 16 dropouts (Figure 3).

**Figure 4:**
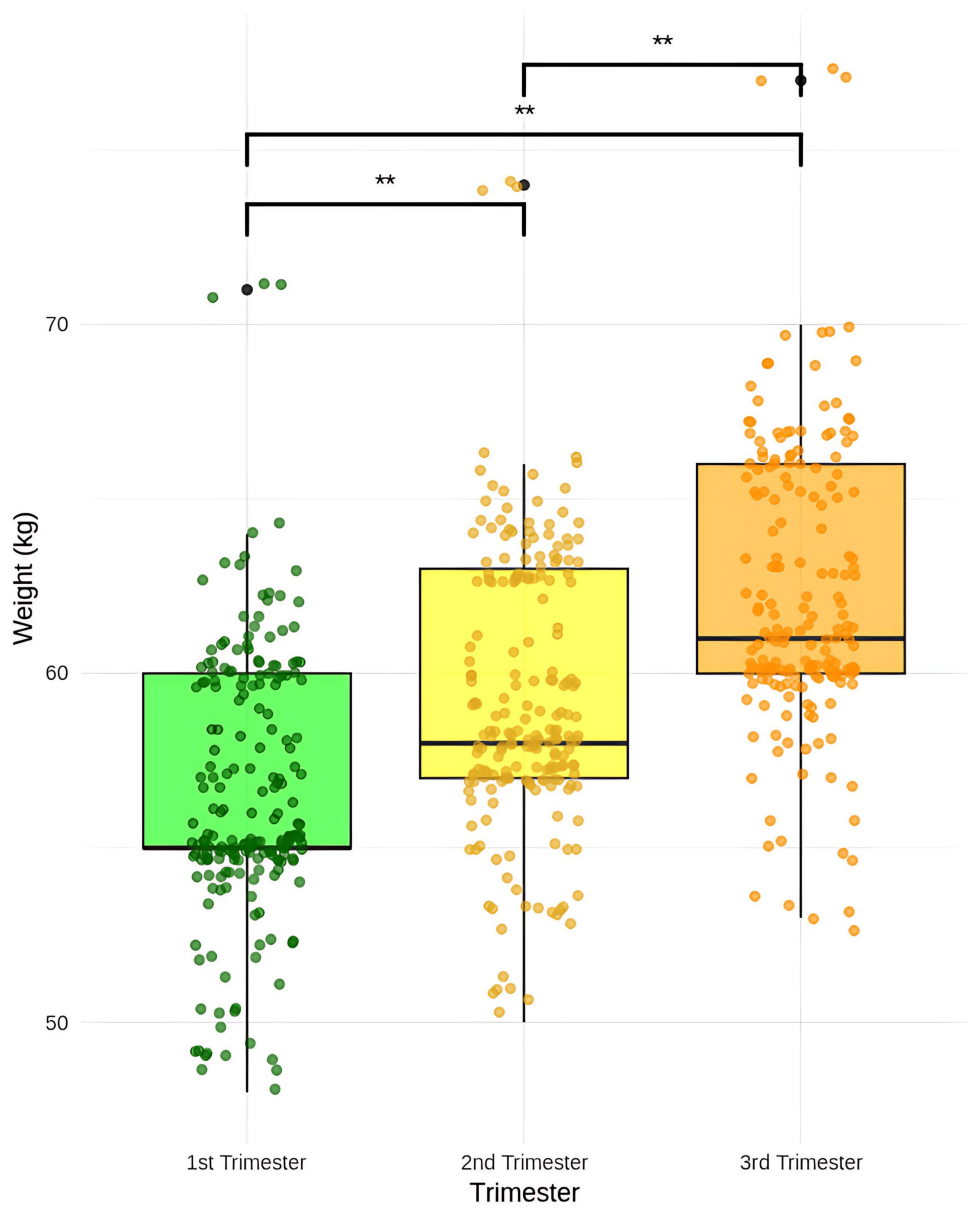
Comparison of weight gain in pregnancy between the first trimester, second trimester, and third trimester dependent t-test, ** p = 0.0001

The first trimester scan was done, along with the dating scan, ranging between six and 10 weeks. The second trimester scan was done on the day of the anomaly scan, around 18-24 weeks. The timing of the third-trimester scan varied among the patients, ranging between 34 and 38 weeks, and was performed along with the growth/term scan. The average time required for ultrasound was around 5-8 minutes, and the clinical parameters were assessed within two minutes. Between the first and second trimesters, the mean weight increased from 56.58 to 59.30 kg, with a mean difference of -2.72, showing a 4.81% change, and the difference was statistically significant (p = 0.0001) (Figure 4).

**Figure 5:**
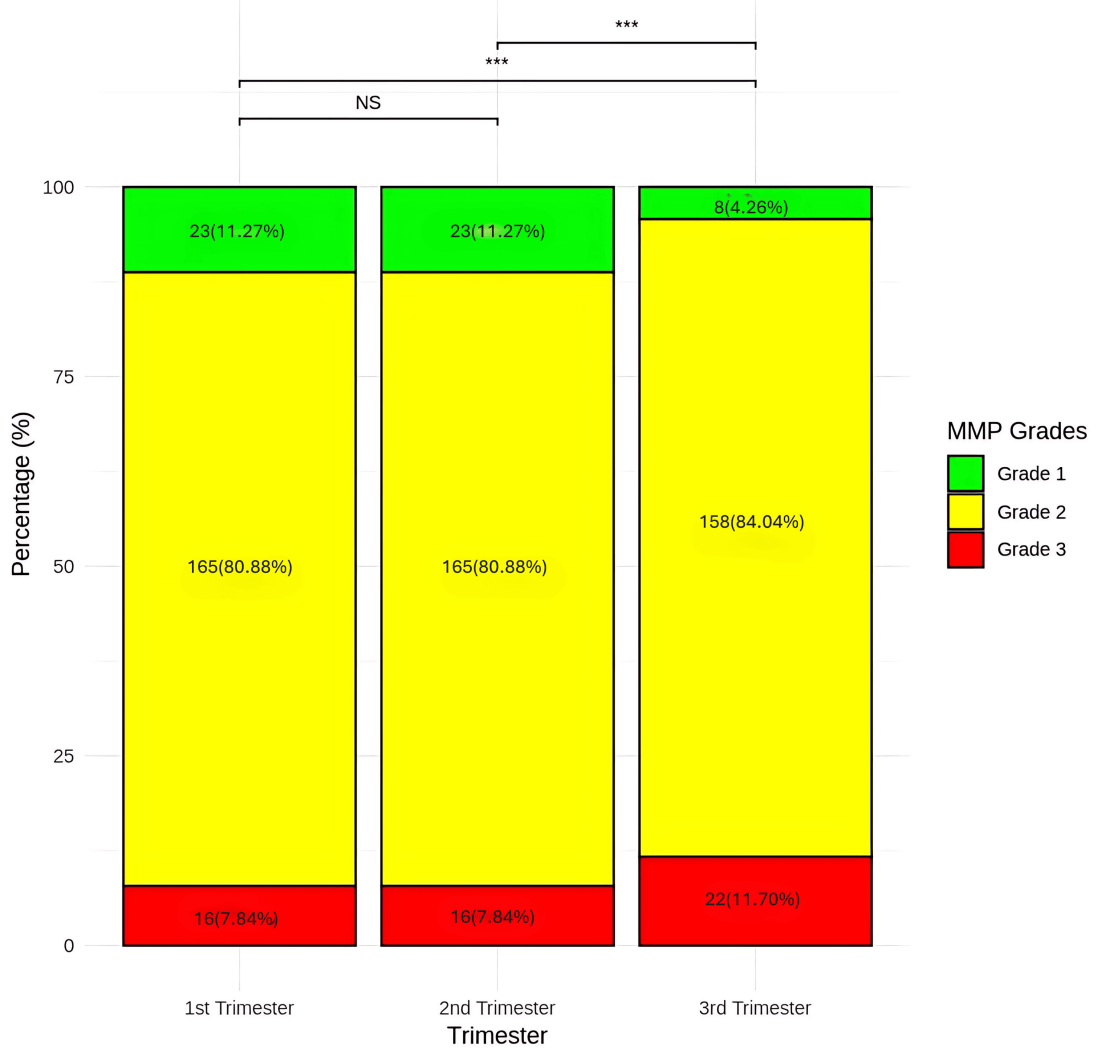
Comparison of MMP grades between the first trimester, second trimester, and third trimester Data presented as number (percentage), Friedman test applied, *** p < 0.001 NS - not significant; MMP - modified Mallampati

Between the first and third trimesters, the mean weight rose from 56.68 to 62.28 kg, with a larger mean difference of -5.60 (9.87% change), also statistically significant (p = 0.0001). Finally, between the second and third trimesters, the mean difference was -2.88, reflecting a 4.85% increase, with statistical significance (p = 0.0001). There was a significant difference in MMP grades across the trimesters (p = 0.0001) (Figure 5).

Pairwise comparisons using the Wilcoxon-matched pairs test revealed no significant difference between the first and second trimesters, as none of the patients had a change in MMP grades (p = 1.0000). However, significant differences were found between the first and third trimesters (p = 0.0001) and between the second and third trimesters when 10.87% of women experienced an increase in one grade (p = 0.0001).

**Figure 6:**
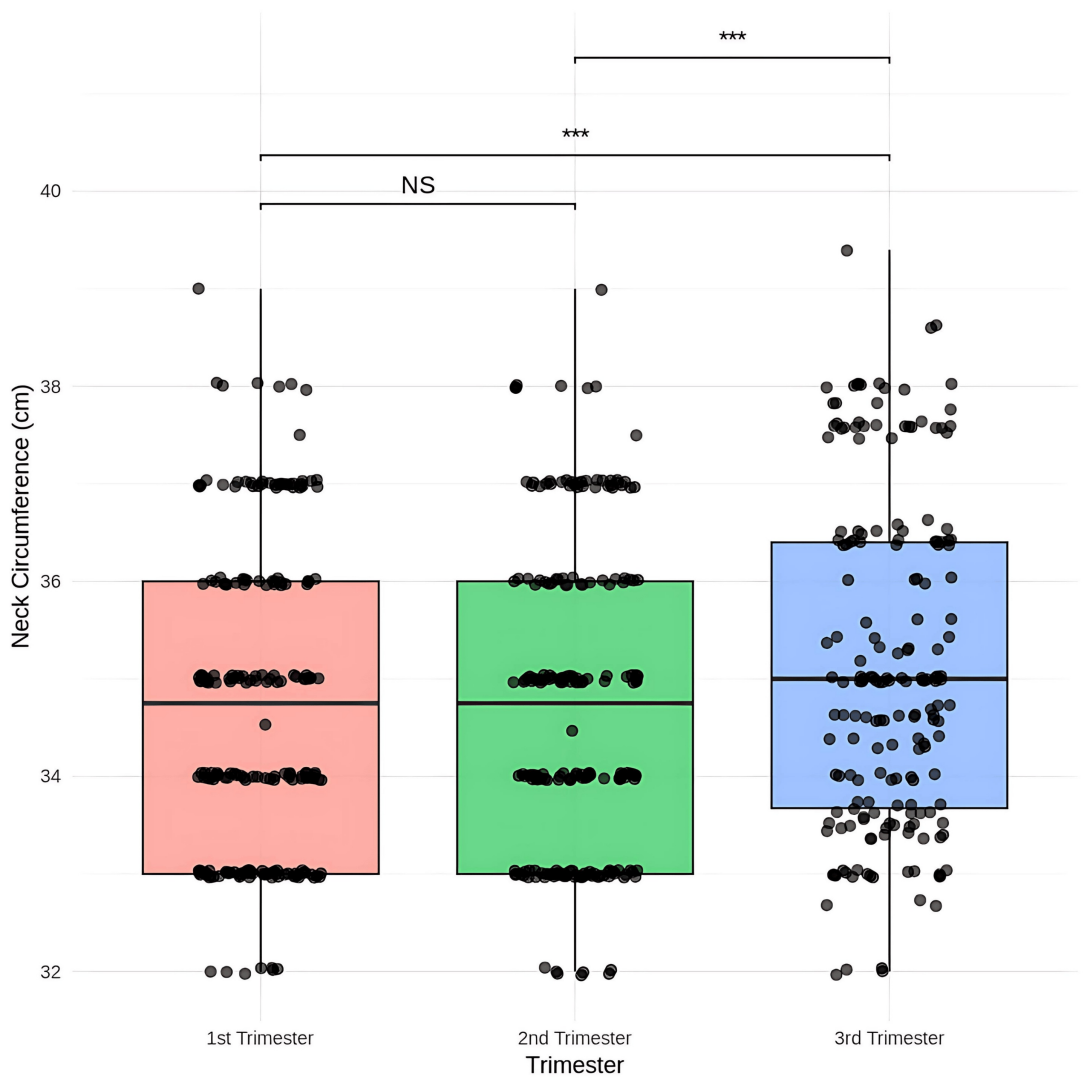
Comparison of the neck circumference scores among first trimester, second trimester, and third trimester Friedman test, post-hoc Wilcoxon signed-rank test with Bonferroni correction, *** p < 0.001 NS - not significant

Statistical analysis showed no significant difference in neck circumference score between the first and second trimesters (p = 1.0000) (Figure 6).

However, there was a significant increase in mean neck circumference measurements from 34.72 cm in the first to 35.13 cm in the third trimester (mean difference = -0.40, p = 0.0001) and from the second (mean = 34.72 cm) to the third trimester (mean difference = -0.40, p = 0.0001).

**Figure 7:**
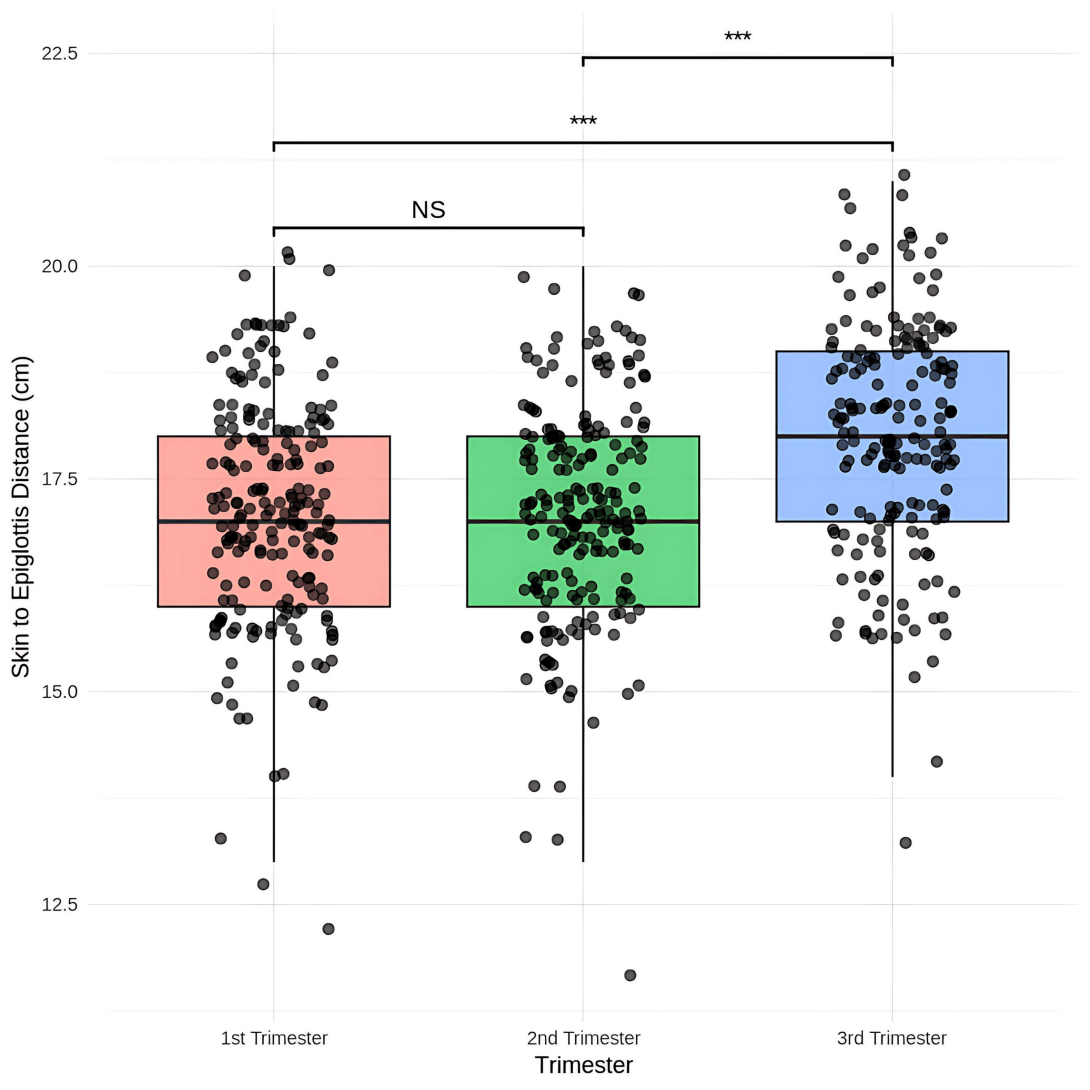
Comparison of the skin-to-epiglottis distance using ultrasonography among the first trimester, second trimester, and third trimester Friedman test, post-hoc Wilcoxon signed-rank test with Bonferroni correction, *** p < 0.001 NS - not significant

Statistical analysis revealed no significant change in the mean skin-to-epiglottis measurements between the first (17.12 mm) and second (17.19 mm) trimesters (p = 1.0000). However, there was a significant increase in measurements from 17.12 mm in the first to 18.01 mm in the third trimester (mean difference = -0.82, p = 0.0001) and from the second to third trimester (mean difference = -0.82, p = 0.0001) (Figure 7).

**Figure 8:**
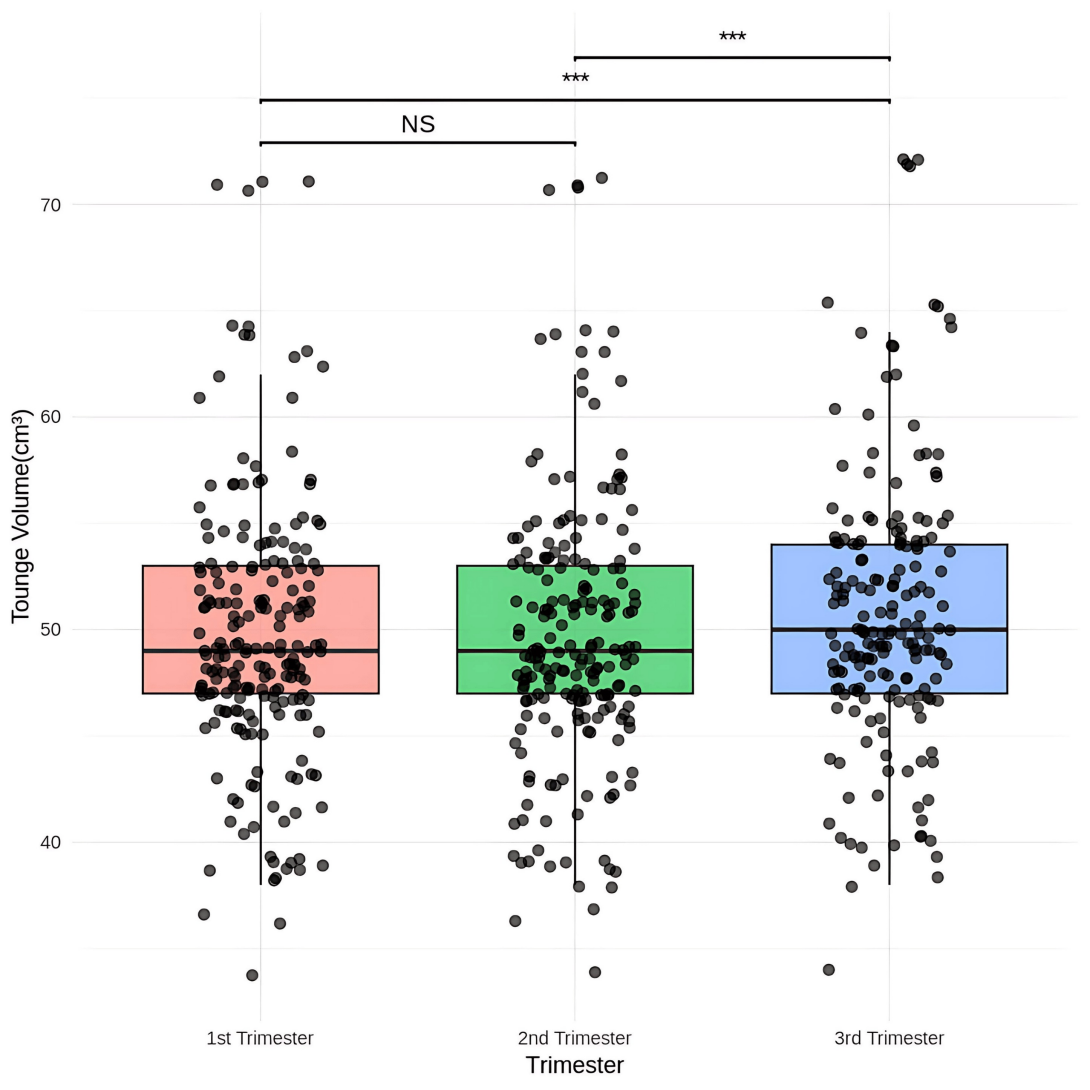
Comparison of the tongue volume scores using ultrasonography among the first trimester, second trimester, and third trimester Friedman test, post-hoc Wilcoxon signed-rank test with Bonferroni correction, *** p < 0.001 NS - not significant

Statistical analysis showed that the mean tongue volume was similar between the first and second trimesters (p = 1.0000). However, there was a significant increase in mean tongue volume from 49.97 mL in the first to 50.80 mL in the third trimester (mean difference = -0.84, p = 0.0001) and from the second to the third trimester (mean difference = -0.84, p = 0.0001) (Figure 8).

The comparison of snoring, voice changes, and facial puffiness across the three trimesters in our study participants revealed significant increases in these parameters with the progress of pregnancy. The incidence of snoring rose from 5.85% in the first trimester to 13.83% in the second and 26.06% in the third (p = 0.0001). Voice changes were absent in the first and second trimesters, but 11.70% of cases were affected in the third trimester (p = 0.0001). Facial puffiness showed a dramatic rise from 0% in the first trimester to 5.85% in the second and 72.34% in the third trimester (p = 0.0001). Significant differences were found in the incidence of snoring and facial puffiness between all the trimesters.

Results obtained by the application of the Cochran Q test to the data in PIH patients indicated significant airway changes (ski-to-epiglottis and tongue volume) among PIH cases (20 cases or 10.58%) across the trimesters (p = 0.0001). Pairwise comparisons using the McNemar test revealed no significant difference between the first and second trimesters (p = 1.0000). However, significant differences were observed between the first and third trimesters (p = 0.0001) and between the second and third trimesters (p = 0.0001).

## Discussion

There are several traditional indices for predicting difficult laryngoscopy, but none of them are 100% sensitive and specific. The evidence for the role of ultrasound in airway assessment is still growing, and the evidence regarding this in pregnancy is sparse [3]. The present study was designed keeping this in mind and with an aim to explore the airway changes in the three trimesters of pregnancy using ultrasound measurements and clinical screening methods, which can help in the prediction of the difficult airway in pregnancy. Significant differences were observed among all the measured airway parameters throughout pregnancy, particularly during the third trimester, with some additional changes in special groups of pregnant women.

Shivashankar et al. examined the effect of pregnancy-related physiological changes on the MMP score. They found that the MMP score tends to increase throughout pregnancy due to weight gain and pharyngeal oedema, which decreases the cross-sectional area of the pharynx [11]. We found similar results in our study. Bala et al., in their study, observed an increase in Mallampati grading among non-pregnant versus normotensive pregnant versus pre-eclamptic women, highlighting a significant rise to Grade IV in the third trimester due to pharyngeal oedema and fatty tissue infiltration [12]. However, we did not encounter Grade IV cases in our group. Additionally, the percentage of women having an increase in airway class (10.87%) is lower compared to the study by Kodali et al., which was done in laboring patients [8].

Mallampati classification is based on the space occupied by the tongue [13]. Bala et al., in their study, observed that an increased tongue volume in pregnant and pre-eclamptic women was associated with a higher risk of difficult intubation [12]. The size of the tongue relative to the oropharyngeal space is a crucial factor in determining how easily the laryngoscope blade can be introduced. The use of ultrasound to calculate the volume of the tongue and further research correlating this value with the Mallampati class could provide more information about the effect of the pregnancy-related changes in the tongue relative to the oropharyngeal space in increasing the Mallampati grades. Various approaches have been used for tongue volume measurement by ultrasonography. In the present study, we used the submandibular ultrasound technique for measuring the tongue volume, and this is said to be a reliable method for airway assessment, even in obese patients, offering good views [10]. We measured the width and cross-sectional area of the tongue to estimate its volume and found that the changes through the course of pregnancy mirrored the increases in the Mallampati grades across the trimesters (p = 0.0001). In a study by Xu et al., tongue thickness was assessed using submental ultrasonography in the median sagittal plane before anaesthesia. They measured the maximum vertical distance from the tongue surface to the submental skin. The researchers found that parturients in the difficult laryngoscopy group had greater tongue thickness measured by ultrasonography. This was found to be an independent predictor for difficult laryngoscopy by multivariate logistic regression, with the odds ratios of 2.554 (95% CI: 1.715-3.802). The authors found the sensitivity and specificity for tongue thickness to be 85% and 91%, respectively, and those for the MMP score to be 73% and 61%, respectively [14]. Nonetheless, tongue thickness was not assessed in our study as the entire volume of the tongue can be affected by pregnancy-related changes.

In one of the early studies on ultrasonographic assessment of the airway, the authors assessed the tongue volume by ultrasound and Mallampati grading in adult patients and found that higher volumes were associated with difficult laryngoscopy grades. However, they did not assess the correlation between the tongue volume and the MMP class [3]. Our study findings show that changes in MMP scoring through the trimesters are concordant with increases in the tongue volume.

In our study, we observed that the neck circumference increased in almost all pregnant patients from the first to the third trimester. Similarly, Bala et al., in their study, observed that an increased neck circumference and chest size in pregnant and pre-eclamptic women [12]. The contributing factors to this could include weight gain during pregnancy and fluid retention and increased soft tissue thickness. Nevertheless, neck circumference is reported as a sensitive predictor of difficult intubation [15].

In our study, we chose the skin-to-epiglottis distance measurements at the level of the thyrohyoid membrane as a measure of the anterior neck soft tissue, and we found a significant increase through the course of pregnancy. In the study by Parameswari et al., while the tongue volume demonstrated reasonable sensitivity and specificity in predicting difficult laryngoscopy, it was not as predictive as the thickness of the anterior neck soft tissue as assessed by the skin to epiglottis distance [3]. Adhikari et al. found that measurements of anterior neck soft tissue thickness at the level of the hyoid bone and thyrohyoid membrane could be used to distinguish easy from difficult laryngoscopy [16]. Similarly, Wu et al., in their study on 203 patients, have shown that the skin-to-hyoid distance, as well as the skin-to-epiglottis distance, were good predictors of difficult laryngoscopy [17].

Increasing maternal age and increasing body weight have been identified as risk factors for the obstetric airway [18]. This means that the changes in the airway progress with increasing body weight over the course of pregnancy. Additionally, the changes will be more in the advanced maternal age. Our study findings showed an increase in airway changes, mainly in the third trimester, corresponding to the increase in weight gain. Additionally, the increases were lower compared to the results of the other studies performed during labor, indicating that the changes are non-linear and increase more after term. We cannot comment on the airway changes with increasing maternal age due to the limited sample in the extremes of age. Increases in the incidence of snoring, voice changes, and facial oedema were the secondary findings of our study, ascertaining the progressive changes in the airway during pregnancy. Anaesthesiologists can encounter pregnant women in various trimesters for obstetric or non-obstetric surgery. Knowledge of the onset and progress of airway changes can help the anaesthesiologist get an idea of when exactly the airway starts becoming difficult during the course of pregnancy and to prepare himself/herself for a difficult airway.

A limitation of the present study is that we did not attempt to correlate the measured ultrasonographic indices with Cormack and Lehane direct laryngoscopy grades, given that most of our patients either had a normal vaginal delivery or caesarean delivery under spinal anaesthesia. Ultrasonographic measurements tend to have interobserver variability. Additionally, we faced a higher rate of dropouts due to loss of follow-up in the third trimester; because in our geographical area, it is a tradition that pregnant females go to their parental home (a different village/city) during the third trimester of pregnancy for labor and delivery. Hence, we could not follow up on these cases. We, however, analyzed the measurements taken in the first and second trimesters in these patients. Nevertheless, more changes in the airway are expected to take place in the third trimester or during labor [8]. Though we collected the data in preeclamptic patients, we did not do a subgroup comparative analysis due to low sample.

## Conclusions

Ultrasonographic measurements of the airway, such as the skin-to-epiglottis distance and tongue volume, increase predominantly in the third trimester of pregnancy. Clinical assessments such as Mallampati scoring and neck circumference measurement coincide with ultrasonographic changes. Furthermore, there is an increasing incidence of a history of snoring, changes in voice, or facial oedema throughout the course of normal singleton pregnancies.
